# Socioeconomic inequalities in allostatic load in a large Norwegian cohort study: the NCDNOR project

**DOI:** 10.1136/jech-2025-225160

**Published:** 2026-04-17

**Authors:** Siri Høivik Storeng, Erik Reidar Sund, Knut Eirik Dalene, Simon Lergenmuller, Inger Ariansen, Steinar Krokstad

**Affiliations:** 1 HUNT Research Centre, Department of Public Health and Nursing, Faculty of Medicine and Health Sciences, Norwegian University of Science and Technology, Trondheim, Norway; 2 Levanger Hospital, Nord-Trøndelag Hospital Trust, Levanger, Norway; 3 Department of Chronic Diseases, Norwegian Institute of Public Health, Oslo, Norway; 4 Method Development and Analytics, Norwegian Institute of Public Health, Oslo, Norway; 5 Cancer Registry of Norway, Department of Registration, Folkehelseinstituttet, Oslo, Norway

**Keywords:** COHORT STUDIES, EPIDEMIOLOGY, Life course epidemiology, SOCIAL CLASS, PUBLIC HEALTH

## Abstract

**Background:**

Allostatic load (AL) is the physiological burden of stress, and it is suggested to be a mediator between socioeconomic position and health. The aim of this article was to study life-course trajectories in levels of AL by employing data collected over three decades (1994–2019) to compare sex and socioeconomic groups in the adult Norwegian population.

**Methods:**

Harmonised data from the following Norwegian health studies were used: the Trøndelag Health Study, the Tromsø Study, the Age 40 Program and CONOR. In total, 264 824 participants aged 30–79 years contributed with 346 312 health examinations. A combined AL score was calculated as the number of biomarkers in the worst quartile among the following eight variables: systolic blood pressure, diastolic blood pressure, resting heart rate, triglycerides, total cholesterol, high-density lipoprotein cholesterol, body mass index and waist-hip ratio. High-sensitivity C reactive protein and glycated haemoglobin were included in sensitivity analyses for a subset of participants. Life-course trajectories were analysed by using a linear mixed model. A shared-parameter model was employed to evaluate attrition caused by death.

**Results:**

Participants with primary education had higher AL compared with secondary and tertiary educated people in all age groups. Men had higher AL than women throughout the life-course. Overall, AL increased with age but levelled off at higher ages among women and decreased at higher ages among men.

**Conclusions:**

We found significant socioeconomic inequalities and sex differences in AL throughout the life-course. This highlights a potential for improvement in public health by effective treatment and public health measures to reduce AL inequities.

**WHAT IS ALREADY KNOWN ON THIS TOPIC**

- Previous studies have found socioeconomic, sex and age differences in allostatic load, but variability in the measurement of allostatic load hampers comparability between studies and there are few longitudinal studies including the whole adult population.

**WHAT THIS STUDY ADDS**

- By using Norwegian population-based health studies including nearly 350 000 health examinations collected over three decades, we are to our knowledge the first to show how life-course trajectories in allostatic load differ by socioeconomic groups.
- By employing a shared-parameter model, we show that the results are not biased by mortality.

**HOW THIS STUDY MIGHT AFFECT RESEARCH, PRACTICE OR POLICY**

- There is potential for improvement in public health through public health policies levelling out socioeconomic inequities, effective treatment of non-communicable diseases and preventive measures to reduce allostatic load.

## Introduction

 Non-communicable diseases (NCDs) are major causes of morbidity and mortality worldwide.^1^ The most prevalent NCDs are cardiovascular disease, cancer, chronic lung disease and diabetes, which combined accounted for 80% of all premature NCD deaths in 2021.^1^ There are complex underlying causes for NCD morbidity and mortality, including a combination of genetic, physiological, environmental, and behavioural factors.^1^ NCDs and their underlying risk factors are unevenly distributed across socioeconomic strata, where low socioeconomic position (SEP) itself constitutes a risk factor for morbidity and mortality.^2^

Stress has been identified as a central pathway from SEP to health outcomes.^3^ Allostatic load (AL) refers to the cumulative physiological burden caused by prolonged exposure or inefficient management of stress.^4^ This could lead to physiological dysregulation and an increased risk of chronic diseases.^4 5^ AL has previously been found to be associated with physical and psychological health, mortality and socioeconomic status.^6–10^ However, variety in the measurement of AL and lack of longitudinal studies hampers comparability between population groups and time periods.^7 10 11^ To our knowledge, no one has studied life-course trajectories in AL stratified by SEP, and few studies have evaluated attrition caused by death. Because data from Norwegian population-based health studies fully represent the non-institutionalised population covering several decades, they are uniquely suited to study life conditions and biological markers in a life-course perspective.^12^ The aim of this study was therefore to use high-quality data from large Norwegian population-based health studies to investigate socioeconomic and sex differences in life-course AL trajectories. Additionally, we employ a shared-parameter model to evaluate attrition caused by death.

## Methods

### Data sources

This paper is a substudy in the project ‘A life-course approach to prevent NCDs in an ageing population–NCDNOR’. NCDNOR is a research project where the aim is to establish new knowledge in the prevention of NCDs by using a concept that includes consolidation of NCD endpoints across somatic disciplines, examining joint and interaction effects of socioeconomic circumstances, life conditions, health behaviours, biological markers and mental health throughout the life-course to identify targets for prevention. It comprises data from major Norwegian population-based health studies and national registers and has been described in detail elsewhere.^12 13^ In this article, we use longitudinal data from survey 2–4 of the Trøndelag Health Study (HUNT Study, 1995–2019), survey 4–7 of the Tromsø Study (1994–2016), the Age 40 Program (1985–1999) and the Cohort of Norway (CONOR, 1994–2003). We obtained data on educational level from the National Educational Database, and data on sex, birth year and mortality from Statistics Norway.

### Study population

In total, 603 662 participants (750 827 health examinations) were eligible for inclusion from the population-based health studies (figure 1). Health examinations with complete information on the eight core AL variables from participants between the ages of 30 and 79 years were included. We excluded health examinations where participants were aged 80 years and older (n=11 058) due to increasing risk of healthy survivor and participant bias.^14^ At the lower age limit (30 years), participants were assumed to have completed their highest degree of education. High-density lipoprotein (HDL) cholesterol and waist-hip ratio were not measured in all parts of the Age 40 Program, and this accounts for a large number of missing values when calculating the AL score (online supplemental table 1). After exclusions, the final sample consisted of 264 824 individuals (52.4% women) including 346 312 health examinations (each participant contributing data from 1 to 4 health examinations between 1994 and 2019).

**Figure 1:**
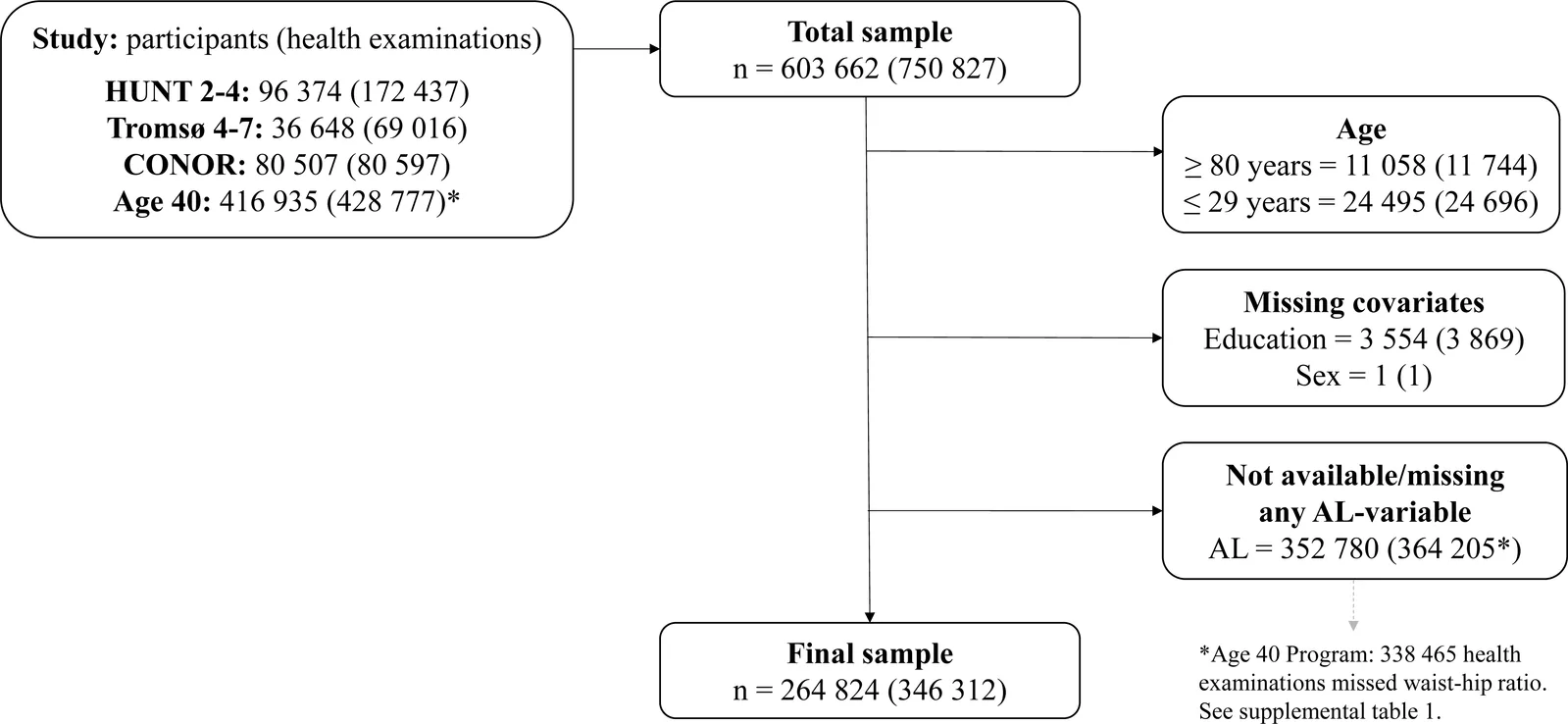
Flow chart of participants included in the study. Age 40, the Age 40 Program; AL, allostatic load; CONOR, Cohort of Norway; HUNT, The Trøndelag Health Study; Tromsø, The Tromsø Study.

### Allostatic load

The variables included in the AL score were mainly cardiovascular and metabolic variables, since these were the available variables from physical measurements and non-fasting blood samples (table 1). This included triglycerides, total cholesterol, HDL cholesterol, resting heart rate, systolic and diastolic blood pressure, body mass index (BMI) and waist-hip ratio. High-sensitivity C reactive protein (CRP) and glycated haemoglobin (HbA1c) were available from a subset of the participants and were included in sensitivity analyses.

**Table 1:** Allostatic load variables, total n=346 312

Variable	Min	Max	Cut-off
Triglycerides (mmol/L)	0.03	29.5	>2.1
Total cholesterol (mmol/L)	2.0	20.0	>6.4
High-density lipoprotein cholesterol (mmol/L)	0	5.2	<1.6
Resting heart rate (bpm)	29	182	>76
Systolic blood pressure (mm Hg)	60	274	>142
Diastolic blood pressure (mm Hg)	30	159	>83
Body mass index (kg/m^2^)	11.9	70.6	>28.9
Waist-hip ratio	0	1.9	>0.9

* Cut-off for allostatic load score

bpm, beats per minute; kg/m2, kilograms per heigt in meters2; mm Hg, millimetres of mercury; mmol/L, millimoles per liter.

The AL score was constructed by dichotomising the eight core biomarkers at the highest quartile from the whole study population including both sexes (>75th percentile = ‘at risk’) and adding the number of ‘at risk’ variables to a total AL score.^11 15^ HDL cholesterol was reversed where the lowest quartile (<25th percentile) was defined as ‘at risk’. The AL score ranged from 0 to 8, with 8 reflecting the highest AL. Several sensitivity analyses were performed to see how alternative AL score calculations affected the results.^15^

### SEP, sex and birth cohort

We analysed AL according to SEP, sex and birth cohort. Highest attained education (registered by 2019) was employed as a time-invariant proxy for SEP and was split into three categories: (1) primary education (0–10 years), (2) secondary education (11–13 years) and (3) tertiary education (higher education). Sex was included as a binary variable (men/women). Birth cohorts were included to investigate temporal changes in AL and split into 10-year bands from 1930 to 1970. The cohorts born before 1930 (29 851 health examinations) and after 1970 (27 673 health examinations) were collapsed due to sparse data.

### Statistical methods

We used a linear mixed model with random intercept to construct life-course trajectories in AL. A linear model was chosen based on the assumption that AL represents a continuously increasing physiological burden, ease of interpretation and satisfactory model fit to the data. The mixed model used all health examinations with complete information on the eight core AL variables at various time points from different individuals. Analyses were adjusted for age, sex, education and birth cohort. The fully adjusted model included age^2^ (which gave best fit to the data) and interactions between age and sex, and age and education. Health behaviours (smoking, physical activity and alcohol consumption) were considered mediators in the SEP-AL pathway and therefore not adjusted for in the model. We used robust estimates of variance and evaluated models according to fit statistics (Akaike and Bayesian information criterion, log likelihood and likelihood ratio tests). To compare cohort differences, we ran separate unadjusted mixed models with random intercepts for each birth cohort using restricted cubic splines with 3 knots for age. To evaluate attrition caused by death, we ran a shared-parameter model combining a mixed model and a survival model.^16^ The following sensitivity analyses were performed in the calculation of the AL score: sex-specific cut-offs, clinical cut-offs, including those taking blood pressure medications in the risk group for elevated systolic and diastolic blood pressure, and lastly analyses stratified by participation year. All analyses were performed in Stata MP V.18. See online supplemental file 1 for a further description of statistical methods and model considerations.

## Results

Table 2 shows demographics by participation period and overall. Due to the inclusion of the Age 40 Program, there was a higher proportion of health examinations in the age group 40–49 years and birth cohort 1950–1959. Educational levels increased over time, and the proportion registered with tertiary education increased from 24.7% to 37.7% from before year 2000 to after year 2010. Overall, 48.8% of the participants were registered with secondary education. The AL score was 0 in 23.9% of the health examinations, meaning that none of the AL variables fell into an ‘at risk’ quartile. There were few health examinations with AL score 5 or higher (<5%).

**Table 2:** Descriptives by participation period, 264 824 individuals and 346 312 health examinations

	Participation period (year)			
	<1999	2000–2009	>2010	Total
	169 912 (49.1%)	113 928 (32.9%)	62 472 (18.0%)	346 312 (100.0%)
Age group (years)				
30–39	18 559 (10.9%)	18 022 (15.8%)	6213 (9.9%)	42 794 (12.4%)
40–49	110 515 (65.0%)	30 783 (27.0%)	14 434 (23.1%)	155 732 (45.0%)
50–59	14 582 (8.6%)	20 960 (18.4%)	16 041 (25.7%)	51 583 (14.9%)
60–69	13 279 (7.8%)	25 409 (22.3%)	15 651 (25.1%)	54 339 (15.7%)
70–79	12 977 (7.6%)	18 754 (16.5%)	10 133 (16.2%)	41 864 (12.1%)
Sex				
Men	79 850 (47.0%)	55 977 (49.1%)	29 134 (46.6%)	164 961 (47.6%)
Women	90 062 (53.0%)	57 951 (50.9%)	33 338 (53.4%)	181 351 (52.4%)
Highest education				
Primary	42 196 (24.8%)	26 969 (23.7%)	9086 (14.5%)	78 251 (22.6%)
Secondary	85 695 (50.4%)	53 505 (47.0%)	29 842 (47.8%)	169 042 (48.8%)
Tertiary	42 021 (24.7%)	33 454 (29.4%)	23 544 (37.7%)	99 019 (28.6%)
Birth cohort				
1880–1929	18 021 (10.6%)	11 830 (10.4%)	0 (0.0%)	29 851 (8.6%)
1930–1939	13 351 (7.9%)	15 277 (13.4%)	1468 (2.3%)	30 096 (8.7%)
1940–1949	15 247 (9.0%)	26 882 (23.6%)	12 258 (19.6%)	54 387 (15.7%)
1950–1959	115 803 (68.2%)	24 848 (21.8%)	15 976 (25.6%)	156 627 (45.2%)
1960–1969	7490 (4.4%)	23 992 (21.1%)	16 196 (25.9%)	47 678 (13.8%)
1970–2000	0 (0.0%)	11 099 (9.7%)	16 574 (26.5%)	27 673 (8.0%)
Allostatic load score				
0	44 003 (25.9%)	26 165 (23.0%)	12 520 (20.0%)	82 688 (23.9%)
1	40 330 (23.7%)	25 873 (22.7%)	13 278 (21.3%)	79 481 (23.0%)
2	30 112 (17.7%)	22 026 (19.3%)	13 152 (21.1%)	65 290 (18.9%)
3	23 369 (13.8%)	17 273 (15.2%)	10 630 (17.0%)	51 272 (14.8%)
4	16 057 (9.5%)	12 034 (10.6%)	7166 (11.5%)	35 257 (10.2%)
5	9299 (5.5%)	6486 (5.7%)	3714 (5.9%)	19 499 (5.6%)
6	4699 (2.8%)	2976 (2.6%)	1509 (2.4%)	9184 (2.7%)
7	1735 (1.0%)	935 (0.8%)	450 (0.7%)	3120 (0.9%)
8	308 (0.2%)	160 (0.1%)	53 (0.1%)	521 (0.2%)
Health study				
Trøndelag Health Study	52 217 (30.7%)	42 186 (37.0%)	42 984 (68.8%)	137 387 (39.7%)
Tromsø Study	6591 (3.9%)	19 124 (16.8%)	19 488 (31.2%)	45 203 (13.1%)
Cohort of Norway	25 353 (14.9%)	51 981 (45.6%)	0 (0.0%)	77 334 (22.3%)
Age 40 Program	85 751 (50.5%)	637 (0.6%)	0 (0.0%)	86 388 (24.9%)

n (%), number of participations (percentage of all participations)

Figure 2 shows the mean level of each risk factor included in the AL score from 30 to 79 years. In general, men had higher risk factor levels until middle ages but then levelled off at a higher rate than for women at higher ages. Systolic blood pressure increased with increasing age for both sexes, but women surpassed men at around 70 years. In levels of total cholesterol, women surpassed men at around 50 years and continued to rise, while it levelled off for men. Triglyceride levels increased until around 40 years for men before they decreased, while there was a continuous increase between 30 and 79 years for women. Waist-hip ratio increased with age for both men and women.

**Figure 2:**
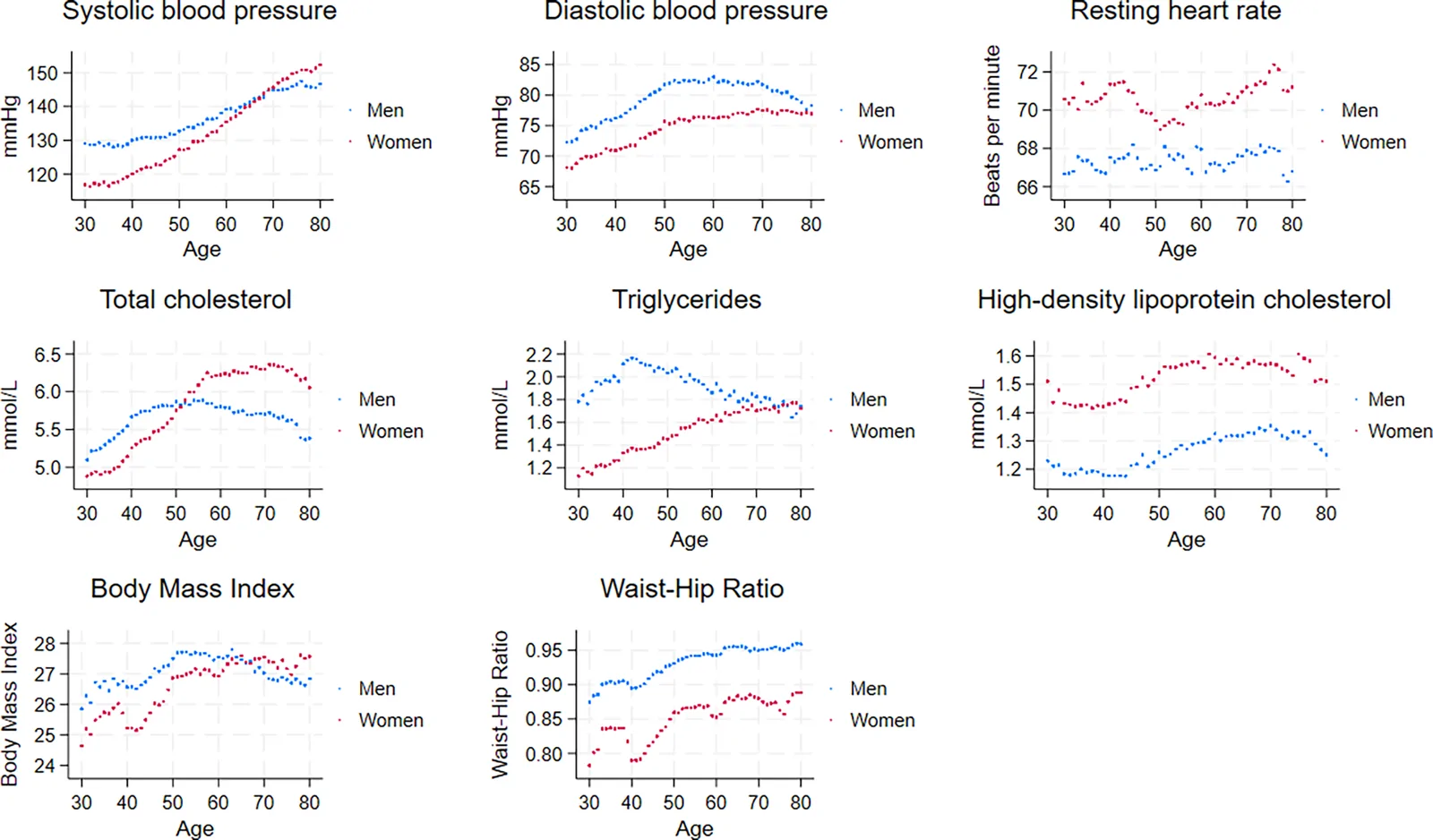
Mean level of each risk factor included in the allostatic load score from 30 to 79 years. mmHg, millimetres of mercury; mmol/L, millimoles per liter

Cut-offs and the proportion of participants with ‘at risk’ values for the AL variables by sex and age group are shown in online supplemental figure 1 and online supplemental table 2. For men, elevated triglycerides and HDL cholesterol were the most prevalent risk factors in the youngest age groups (30–49 years), whereas elevated systolic blood pressure and waist-hip ratio were the most prevalent risk factors in older age groups (60–79 years). For women, elevated resting heart rate and BMI were the most prevalent risk factors in younger age groups (30–49 years), whereas elevated total cholesterol and systolic blood pressure were most prevalent among the older age groups (60–79 years).

Table 3 shows results from unadjusted and adjusted mixed models compared with the shared-parameter model accounting for mortality. Estimates from the adjusted mixed model showed that women had −0.84 lower AL score than men. Online supplemental figure 2 shows that men had higher AL scores compared with women throughout the life-course, but AL decreased among men after 60 years, whereas it levelled off for women. Those with secondary and tertiary education had −0.17 and −0.39 lower AL score compared with the group with primary education. There was a significant interaction effect between age and education, and age and sex. Those with higher education and women had a smaller increase in AL over the life-course (age-covariate interaction), and men and those with higher education had a steeper dip in older age (age^2^–covariate interaction) compared with primary educated and women. The results from the mixed model were similar to the shared-parameter model (main estimates illustrated in online supplemental figure 3). Each unit increase in the baseline AL score was associated with death with an HR of 1.14 (loading factor, shared-parameter model). Model fit was better for the mixed model compared with the shared-parameter model (online supplemental table 3).

**Table 3:** Associations between age, sex, education, birth cohort and allostatic load from unadjusted and adjusted mixed models, shared-parameter and Weibull survival model. In total 264 824 individuals, 346 312 health examinations and 43 715 deaths.

	Unadjusted mixed model		Adjusted mixed model		Shared-parameter model	
	Coefficient	95% CI	Coefficient	95% CI	Coefficient	95% CI
Mixed model						
Random effects						
Participant variance (intercept)	1.44	(1.43 to 1.46)	1.22	(1.20 to 1.23)	1.22	(1.20 to 1.23)
Residual variance	1.28	(1.27 to 1.30)	1.25	(1.24 to 1.26)	1.25	(1.24 to 1.26)
Fixed effects						
Intercept	0.92	(0.90 to 0.93)	2.10	(2.04 to 2.15)	2.09	(2.04 to 2.15)
Age	0.071	(0.069 to 0.072)	0.100	(0.095 to 0.104)	0.098	(0.094 to 0.103)
Age^2^	−0.00070	(−0.00073 to 0.00067)	−0.0017	(−0.0018 to −0.0016)	−0.0016	(−0.0017 to 0.0016)
Sex						
Men			Ref.		Ref.	
Women			−0.84	(−0.87 to −0.81)	−0.84	(−0.88 to −0.81)
Education						
Primary			Ref.		Ref.	
Secondary			−0.17	(−0.22 to −0.12)	−0.17	(−0.22 to −0.12)
Tertiary			−0.39	(−0.44 to −0.34)	−0.39	(−0.44 to −0.34)
Birth cohort						
1880–1920			Ref.		Ref.	
1930–1939			−0.29	(−0.32 to −0.26)	−0.29	(−0.32 to −0.26)
1940–1949			−0.41	(−0.44 to −0.38)	−0.40	(−0.43 to −0.37)
1950–1959			−0.61	(−0.64 to −0.58)	−0.61	(−0.64 to −0.57)
1960–1969			−0.52	(−0.55 to −0.48)	−0.51	(−0.55 to −0.48)
1970–2000			−0.30	(−0.34 to −0.26)	−0.29	(−0.33 to −0.25)
Interactions						
Education*age						
Primary			Ref.		Ref.	
Secondary			−0.009	(−0.013 to −0.004)	−0.008	(−0.013 to −0.004)
Tertiary			−0.026	(−0.031 to −0.021)	−0.025	(−0.030 to −0.020)
Education*age^2^						
Primary			Ref.		Ref.	
Secondary			0.0002	(0.0001 to 0.0003)	0.0001	(0.0001 to 0.0002)
Tertiary			0.0005	(0.0004 to 0.0006)	0.0005	(0.0004 to 0.0006)
Sex*age						
Men			Ref.		Ref.	
Women			−0.022	(−0.025 to −0.018)	−0.021	(−0.025 to −0.018)
Sex*age^2^						
Men			Ref.		Ref.	
Women			0.0008	(0.0007 to 0.0009)	0.0008	(0.0007 to 0.0008)
Weibull survival model*			HR†		HR‡	
Sex						
Men			Ref.		Ref.	
Women			0.67	(0.66 to 0.68)	0.67	(0.65 to 0.68)
Education						
Primary			Ref.		Ref.	
Secondary			0.85	(0.83 to 0.86)	0.85	(0.83 to 0.87)
Tertiary			0.68	(0.66 to 0.70)	0.69	(0.67 to 0.71)
Weibull shape parameter (ln(p))			2.32	(2.31 to 2.32)	2.32	(2.31 to 2.33)
Intercept			2.22e–20	(1.64e–20 to 3.00e–20)	1.85e–20	(1.36e–20 to 2.50e–20)
Random intercept loading factor (U0)					1.14	(1.13 to 1.16)

* Weibull survival model includes 264 824 participants and 43 715 deaths.

† Separate Weibull survival model.

‡ Weibull survival model in the shared-parameter model.

CI, confidence interval; Ref., reference category.

Figure 3 shows life-course trajectories in AL for SEP-groups separately for men and women aged 30–79 years. This illustrates that educational inequalities were largest in the middle-aged groups and smaller at younger and higher ages. Online supplemental figure 4 shows separate AL trajectories for the birth cohorts and mean AL for men and women from 30 to 79 years. In general, the birth cohort trajectories follow the mean AL, but there is more variation among the female birth cohorts compared with male.

**Figure 3:**
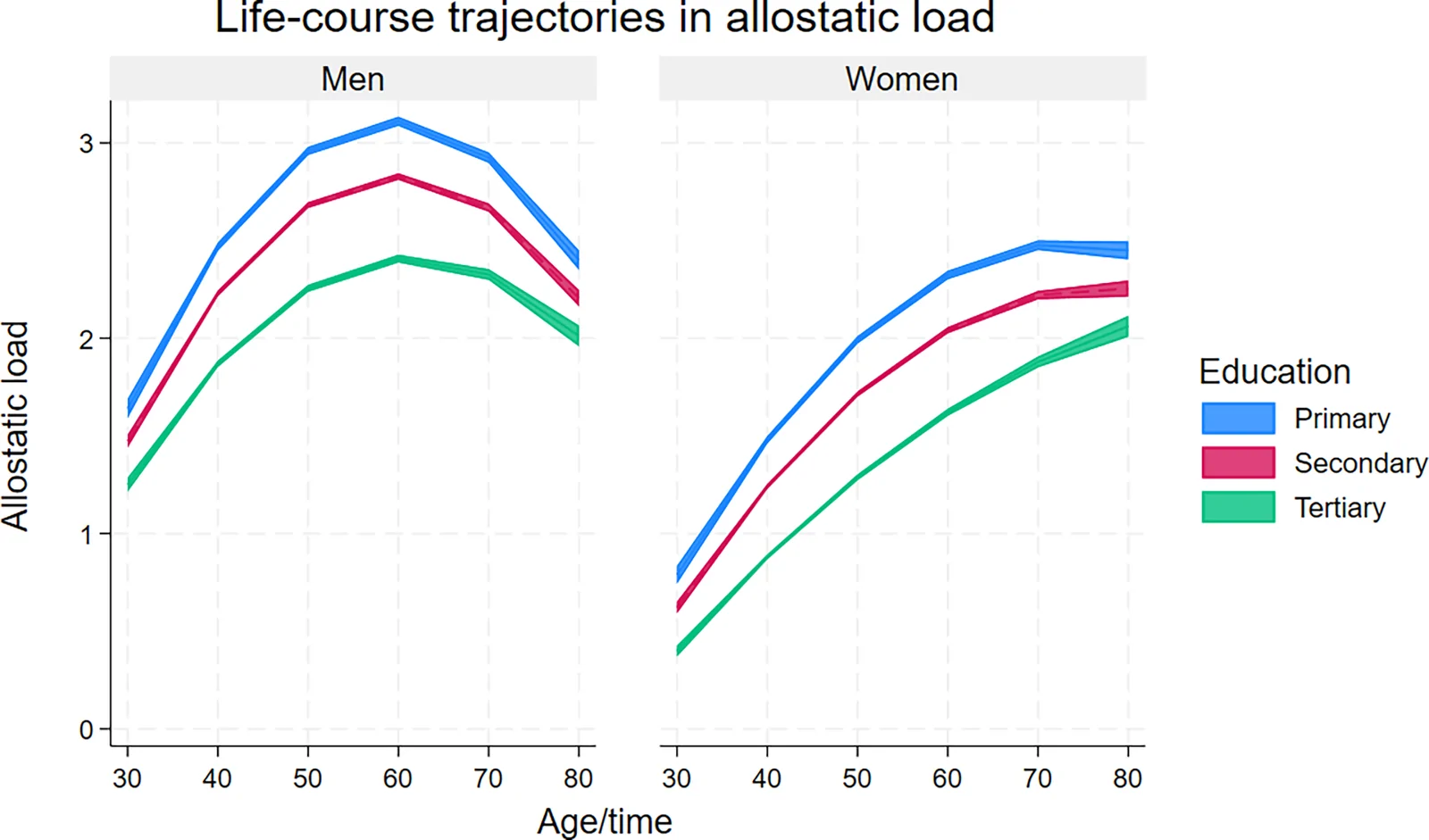
Life-course trajectories in allostatic load by sex and education.

## Discussion

### Key findings

In this study, we found significant differences in AL by SEP and sex throughout the life-course. We found that men had higher AL compared with women and that those with lower education had higher AL compared with higher educated groups. We found that socioeconomic inequalities in AL were largest in the middle-aged groups and smaller at younger and higher ages.

### Discussion of results

Overall, previous systematic reviews find that men have higher AL compared with women.^17 18^ This is in line with our results. However, Yang and Kozloski show how both AL-life-course trajectories and sex differences in AL may be influenced by the inclusion of different biomarkers in the AL score.^19^ Due to known sex differences in the stress response and biomarkers,^20^ we also calculated a sex-specific AL score with separate 75th risk percentiles for men and women. This revealed the same general trends in AL over the life-course, but men’s curve was shifted downwards (AL scores ranging from 1 to 2.5), whereas women showed a steeper increase during the life-course (AL scores ranging from 0.5 to 3). In their selective review, Juster and colleagues highlight the importance of taking gender into account when studying social determinants of health.^20^ Unfortunately, we did not have enough information to include this in the current study.

Our finding that lower socioeconomic groups have higher AL is in line with previous systematic reviews and one meta-analysis.^6 8 21^ We found that socioeconomic inequalities in AL were largest in the middle-aged groups and smaller at younger and higher ages. A similar educational pattern has been found in Scotland.^22^ This may suggest that the effects of education on the exposure and bodily response to chronic stress increase until middle age, whereas in older ages other factors may play a greater role (eg, genetics, environmental/behavioural factors). In our analyses, we chose education as a proxy for SEP based on the assumption that it was stable from 30 years of age. However, it is important to note that the educational level in the study population increased over time, and that a low, moderate and high education level could have slightly different effects across cohorts. Further, education may represent a different dimension of socioeconomic status compared with income or occupation.

Regarding the age patterns, we found that AL increased from 30 to 60 years in both men and women and thereafter flattened out for women and decreased among men until 80 years. This resulted in similar levels of AL for men and women at old age. Horizontal AL trajectories at older ages have also been found in the USA,^23^ as well as decreasing AL at higher ages among men in China.^24^ Even though the prevalence of chronic diseases increases with age (which could lead to stress and higher AL scores), older people may have better coping strategies than younger individuals.^17^ Yang and Kozloski found a similar pattern as ours with decreasing AL scores at higher ages for men when including metabolic/cardiovascular markers (waist-hip ratio, systolic blood pressure, diastolic blood pressure, triglycerides, HDL cholesterol, BMI, HbA1c and glucose), but that inflammatory markers (CRP, fibrinogen and albumin) showed a male advantage throughout life.^19^ This illustrates how inclusion of different markers in the AL score as well as sex differences may affect the life-course trajectories. Healthy participant bias could also contribute to this finding, where men have lower participation rates compared with women and participants have been found to be healthier than non-participants.^14 25 26^

### Strengths and limitations

Strengths of this study are that we employ high-quality data from large Norwegian population-based health studies over three decades. We study life-course trajectories in AL stratified by SEP and sex, which few studies have done before. An identically constructed measure of AL based on a representative adult population spanning three decades enables valid comparisons of groups and changes over the life-course, which has been a significant limitation in previous research. Additionally, results from the shared-parameter model allowed us to evaluate attrition caused by death, which is essential considering AL’s association with mortality.^7^

One limitation in this research field is that AL is a complex clinical construct with heterogeneity in operationalisation and no gold standard of measurement.^6 8 10^ A meta-analysis from 2023 including 13 studies found a variety of 40 biomarkers from 12 different physiological systems used to construct the AL score, and that researchers tended to use what information is available to them to define AL.^10^ Some biomarkers are more frequently used than others, and individual biomarkers vary in predicting health outcomes and mortality.^10 27^ However, the sum of biological markers in AL has been found to better predict mortality and physical outcomes than individual markers,^27 28^ supporting the use of AL to measure a total ‘load’.

A limitation in this study was that the variables used to construct the AL score are mainly cardiovascular/metabolic markers. However, we were also able to conduct sensitivity analyses with CRP and HbA1c included in the AL score for a subset of the study population (online supplemental table 1 and online supplemental figure 5). These analyses showed similar trends overall, as well as inequalities in SEP and sex differences in AL over the life-course, supporting the main findings in this study.

In addition to heterogeneity in definitions of AL, there also exist several ways of calculating the AL score.^15^ In this article, we have employed the most common method, which is to calculate the AL score based on falling into the worst quartile of each risk factor in the whole study population. Downsides of this method are that the cut-offs will be affected by the age distribution in the study population, and by the prevalence of the risk factor—which may change over time. In the present study, the study sample was dominated by 40-year-olds, and measurements were taken over a long time period, from 1994 to 2019. The latter may mask time trends, which we investigated in a sensitivity analysis. Online supplemental figure 6 shows AL trajectories from 30 to 79 years by participation period, and depicts a higher AL score among those aged 50–79 who participated before 1999 compared with between 2000–2009 and after 2010.

We did several sensitivity analyses to see how the choice of AL-calculation method affected the results, and they gave similar results (online supplemental figure 5). In review studies, the choice of method has been found to have a modest impact on explained variance and prediction of various health outcomes.^11 15 29^ Carbone and colleagues also note that a longitudinal data design including the same measurement method of AL over time will be less sensitive to the choice of AL score calculation.^11^ The cut-offs in this study (see online supplemental table 2) were in line with clinically defined treatment boundaries (clearly defined for; blood pressure 140/90 mm Hg, total cholesterol 7.0 mmol/L, and waist-hip ratio <0.85 for women and <0.9 for men, with recommendations for; HDL cholesterol >1.0 mmol/L for men and >1.3 mmol/L for women, resting heart rate <80 bpm, and triglycerides <2.0 mmol/L), except for BMI due to high prevalence of obesity in the Norwegian population. Thus, there is a clinical relevance of being defined in the risk group for each biomarker (highest 75th percentile) in the AL score.

It is debated whether those reporting using medications (eg, blood pressure and cholesterol lowering medicines) should be included in the risk group when calculating AL.^15^ We chose to include variables in the AL score irrespective of medication usage because we wanted to capture the physiological stress reflected at that moment. However, taking medications could mask underlying bodily exposure to chronic stress due to established disease despite ‘normal’ measurements. Therefore, as a sensitivity analysis, we included those taking blood pressure lowering medications in the risk group for diastolic and systolic blood pressure, and this did not alter the main results in this study (see online supplemental figure 5).

### Relevance and implications

The WHO’s Sustainable Development Goal is to reduce premature mortality due to NCDs by 1/3 from 2015 to 2030.^1^ AL reflects the physiological burden of stress as a preclinical stage of disease. Disentangling SEP’s role in the pathway from stress to disease may uncover targets for both treatment and preventive measures to reduce the burden of chronic disease. As noted by Duong and colleagues, the role of stress in the development of disease needs to be considered in both public policies and healthcare practices, targeted both at community and individual levels.^30^ The results from this study show that there is potential for improvement in public health through public health policies levelling out socioeconomic inequities in society and the health services.

## Supplementary material

10.1136/jech-2025-225160 online supplemental figure 1

10.1136/jech-2025-225160 online supplemental figure 2

10.1136/jech-2025-225160 online supplemental figure 3

10.1136/jech-2025-225160 online supplemental figure 4

10.1136/jech-2025-225160 online supplemental figure 5

10.1136/jech-2025-225160 online supplemental figure 6

10.1136/jech-2025-225160 online supplemental file 1

10.1136/jech-2025-225160 online supplemental table 1

10.1136/jech-2025-225160 online supplemental table 2

10.1136/jech-2025-225160 online supplemental table 3

## Data Availability

Data may be obtained from a third party and are not publicly available.
