# Supplementary material for: Socioeconomic inequalities in allostatic load in a large Norwegian cohort study: the NCDNOR project
Source: J Epidemiol Community Health. 2026 Apr 17;80(11):e225160. doi: 10.1136/jech-2025-225160 (PMC13691683; doi:10.1136/jech-2025-225160)
Supplement: Supplementary file 1 [file jech-80-11-s007.pdf]

Supplemental file 1. Methodological considerations

All analyses were performed in Stata MP version 18

Mixed model

Model considerations [1]:

- Participation age was used as timeline. Different transformations of age were compared (linear, squared, piecewise linear splines). Based on model fit and parsimony, age and age^2 were included in the model.
- Sex, education, and birth cohort entered as categorical variables.
- Interaction between age*sex and age*education were included based on a priory knowledge. Squared interaction terms were included to allow the curvature to differ between sexes and educational levels.
- A mixed model with random slopes showed better fit than a model with only a random intercept (compared with LR-test), however there was very little variance explained by adding random slopes (0,000064 (95% CI: 0,000055-0,000076)). In addition to having a complex overall model (non-convergence was a problem for the shared-parameter model especially) a mixed model with random intercept only was chosen.
- The postestimation command "*margins*" (and accompanying "*marginsplot*") in Stata was used to obtain figure for AL trajectories by SEP-groups separately for men and women (calculated at 10-year age intervals; 30, 40, 50, 60, 70, 80).

Shared-parameter model

Model considerations [2, 3]:

- AL has been found to be associated with mortality [4], but effect estimates could be affected if those with the highest AL-score are lost to follow-up due to higher risk of mortality. Therefore, to evaluate this bias we ran a shared-parameter model consisting of a linear mixed model linked to a Weibull survival model through a shared latent random effect variable (intercept only). In other words, the inclusion of a survival model "adjusts" the original model for mortality enabling us to evaluate how this

affects the estimates and model fit. The loading factor (U0) in the shared-parameter model describes the association between the subject-specific latent baseline AL-score and mortality hazards [3]. U0 was constricted to 1 in the mixed model but allowed to vary freely in the survival model enabling evaluation of survivor bias. In the Weibull survival model, age was used as timescale and education and sex were included as covariates. See Griswold et al. (2021) for further information on the shared-parameter methodology and examples of coding [3].

- The survival model was a Weibull survival model using age as timeline and adjusted for sex and education. We chose to retain a simple survival model, since the focus was to include this as an "adjustment" model to see how the estimates of the linear mixed model were affected.

References

1 Rabe-Hesketh S, Skrondal A. *Multilevel and Longitudinal Modeling Using Stata*. Texas: Stata Press 2022.

2 Yildirim E, Karasoy D. gsem: A Stata command for parametric joint modelling of longitudinal and accelerated failure time models. *Comput Methods Programs Biomed* 2020;**196**:105612.

3 Griswold ME, Talluri R, Zhu X*, et al.* Reflection on modern methods: shared-parameter models for longitudinal studies with missing data. *Int J Epidemiol* 2021;**50**:1384–93.

4 Parker HW, Abreu AM, Sullivan MC*, et al.* Allostatic Load and Mortality: A Systematic Review and Meta-Analysis. *Am J Prev Med* 2022;**63**:131–40.
