# Supplementary material for: Socioeconomic inequalities in allostatic load in a large Norwegian cohort study: the NCDNOR project
Source: J Epidemiol Community Health. 2026 Apr 17;80(11):e225160. doi: 10.1136/jech-2025-225160 (PMC13691683; doi:10.1136/jech-2025-225160)
Supplement: Supplementary file 2 [file jech-80-11-s008.pdf]

Supplemental table 1. Observed and missing allostatic load variables, total n=710 517 health examinations

Variable	Observed n	Observed %	Missing n	Missing %	Missing pattern n (%)	Missing pattern n (%)	Missing pattern n (%)
Triglycerides (mmol/L)	707 356	99.6	3161	0.4	x	x	x
Total cholesterol (mmol/L)	706 740	99.5	3777	0.5	x	x	x
High-density lipoprotein cholesterol (mmol/L)	407 831	57.4	302 686*	42.6	x	-	x
Resting heart rate (bpm)	708 364	99.7	2153	0.3	x	x	x
Systolic blood pressure (mmHg)	707 886	99.6	2631	0.4	x	x	x
Diastolic blood pressure (mmHg)	707 875	99.6	2642	0.4	x	x	x
Body Mass Index (kg/m²)	707 263	99.5	3254	0.5	x	x	x
Waist-Hip Ratio	351 273	49.4	359 244*	50.6	x	-	-
**Total**	346 312	205.2	364 205	51.3	346 312 (49%)	296 280 (42%)	58 965 (8%)
**Sensitivity analyses**							
High sensitivity C-reactive protein (mg/L)	133 905	18.8	576 612	81.2			
Glycated haemoglobin (mmol/mol)	89 350	12.6	621 167	87.4			

*In the Age 40 Program, 297 645 participants had missing high-density lipoprotein cholesterol values, and 338 465 participants were missing waist-hip ratio measurements
x = observed, - = missing
