# Supplementary material for: Socioeconomic inequalities in allostatic load in a large Norwegian cohort study: the NCDNOR project
Source: J Epidemiol Community Health. 2026 Apr 17;80(11):e225160. doi: 10.1136/jech-2025-225160 (PMC13691683; doi:10.1136/jech-2025-225160)
Supplement: Supplementary file 3 [file jech-80-11-s009.pdf]

Supplemental table 2. Cut-offs for allostatic load-variables, with number and percentage of age group in risk group for men (n=164 961) and women (n=181 351)

	Age group (years)											
	30-39		40-49		50-59		60-69		70-79		Total	
**Men**	19 329 (11.7%)		72 248 (43.8%)		24 770 (15.0%)		27 480 (16.7%)		21 134 (12.8%)		164 961 (100.0%)	
	Cut-off	Risk (%)	Cut-off	Risk (%)	Cut-off	Risk (%)	Cut-off	Risk (%)	Cut-off	Risk (%)	Cut-off	Risk (%)
Triglycerides (mmol/L)	2.4	6 094 (31.5%)	2.6	27 512 (38.1%)	2.4	8 205 (33.1%)	2.3	8 031 (29.2%)	2.2	5 525 (26.1%)	2.4	55 367 (33.6%)
Total cholesterol (mmol/L)	6.0	2 928 (15.1%)	6.4	17 540 (24.3%)	6.5	6 918 (27.9%)	6.5	7 345 (26.7%)	6.4	5 221 (24.7%)	6.4	39 952 (24.2%)
High-density lipoprotein cholesterol (mmol/L)	1.4	8 614 (44.6%)	1.4	32 167 (44.5%)	1.5	9 066 (36.6%)	1.5	8 809 (32.1%)	1.5	6 987 (33.1%)	1.4	65 643 (39.8%)
Resting heart rate (bpm)	74	3 640 (18.8%)	74	14 192 (19.6%)	74	5 068 (20.5%)	75	5 650 (20.6%)	76	4 739 (22.4%)	74	33 289 (20.2%)
Systolic blood pressure (mmHg)	137	2 563 (13.3%)	139	13 008 (18.0%)	146	7 643 (30.9%)	154	12 096 (44.0%)	159	11 401 (53.9%)	145	46 711 (28.3%)
Diastolic blood pressure (mmHg)	80	3 005 (15.5%)	84	18 635 (25.8%)	89	10 588 (42.7%)	89	11 600 (42.2%)	87	7 761 (36.7%)	86	51 589 (31.3%)
Body-Mass Index (kg/m^2)	28.5	4 267 (22.1%)	28.7	16 888 (23.4%)	29.7	8 054 (32.5%)	29.5	8 435 (30.7%)	28.9	5 417 (25.6%)	29.0	43 061 (26.1%)
Waist-Hip Ratio	0.93	4 295 (22.2%)	0.94	19 419 (26.9%)	0.98	12 272 (49.5%)	1.0	15 141 (55.1%)	1.0	12 047 (57.0%)	0.96	63 174 (38.3%)
**Women**	23 465 (12.9%)		83 484 (46.0%)		26 813 (14.8%)		26 859 (14.8%)		20 730 (11.4%)		181 351 (100.0%)	
	Cut-off	Risk (%)	Cut-off	Risk (%)	Cut-off	Risk (%)	Cut-off	Risk (%)	Cut-off	Risk (%)	Cut-off	Risk (%)
Triglycerides (mmol/L)	1.5	2 234 (9.5%)	1.6	10 792 (12.9%)	1.9	5 170 (19.3%)	2.0	6 108 (22.7%)	2.1	5 020 (24.2%)	1.8	29 324 (16.2%)
Total cholesterol (mmol/L)	5.6	1 763 (7.5%)	6.0	11 179 (13.4%)	6.8	9 309 (34.7%)	7.0	11548 (43.0%)	7.1	9 271 (44.7%)	6.4	43 070 (23.7%)
High-density lipoprotein cholesterol (mmol/L)	1.6	4 161 (17.7%)	1.7	13 623 (16.3%)	1.8	3 343 (12.5%)	1.8	3 381 (12.6%)	1.8	2 808 (13.5%)	1.7	27 316 (15.1%)
Resting heart rate (bpm)	77	6 284 (26.8%)	78	22 760 (27.3%)	76	6 571 (24.5%)	78	7 301 (27.2%)	79	6 364 (30.7%)	78	49 280 (27.2%)
Systolic blood pressure (mmHg)	125	895 (3.8%)	130	7 383 (8.8%)	142	6 612 (24.7%)	153	11 138 (41.5%)	164	12172 (58.7%)	139	38 200 (21.1%)
Diastolic blood pressure (mmHg)	75	1 777 (7.6%)	78	9 969 (11.9%)	83	6 193 (23.1%)	84	6 870 (25.6%)	85	5 831 (28.1%)	80	30 640 (16.9%)
Body-Mass Index (kg/m^2)	27.7	4 517 (19.2%)	27.6	15 292 (18.3%)	29.6	7 957 (29.7%)	30.0	8 771 (32.7%)	30.1	6 980 (33.7%)	28.7	43 517 (24.0%)
Waist-Hip Ratio	0.86	2 129 (9.1%)	0.85	4 934 (5.9%)	0.92	5 536 (20.6%)	0.93	6 244 (23.2%)	0.93	4 536 (21.9%)	0.89	23 379 (12.9%)
