# Supplementary material for: Socioeconomic inequalities in allostatic load in a large Norwegian cohort study: the NCDNOR project
Source: J Epidemiol Community Health. 2026 Apr 17;80(11):e225160. doi: 10.1136/jech-2025-225160 (PMC13691683; doi:10.1136/jech-2025-225160)
Supplement: Supplementary file 4 [file jech-80-11-s010.pdf]

Supplemental table 3. Fit statistics for unadjusted and adjusted mixed models, Weibull survival model and shared-parameter model

Fit statistics	Unadjusted mixed model	Adjusted mixed model	Weibull survival model	Shared-parameter model	Adjusted mixed + Weibull survival model
**BIC**	1300270	1270497	25482.04	1673850	1295979
**AIC**	1300216	1270292	25429.61	1673581	1295722
**LL**	-650103	-635127.2	-13776.5	-836766	-648904

Abbreviations: AIC = Akaike Information Criterion, BIC = Bayesian Information Criterion, LL = Log Likelihood
